# Prospects for Precision Medicine in Acute Myocardial Infarction: Patient-Level Insights into Myocardial Injury and Repair

**DOI:** 10.3390/jcm12144668

**Published:** 2023-07-13

**Authors:** Mohammad Alkhalil, Giovanni Luigi De Maria, Naveed Akbar, Neil Ruparelia, Robin P. Choudhury

**Affiliations:** 1 Cardiothoracic Centre, Freeman Hospital, Newcastle-upon-Tyne NE7 7DN, UK; mak-83@hotmail.com; 2 Translational and Clinical Research Institute, Newcastle University, Newcastle-upon-Tyne NE1 7RU, UK; 3 Cardiology Department, John Radcliffe Hospital, Oxford OX3 9DU, UK; 4 Division of Cardiovascular Medicine, Radcliffe Department of Medicine, University of Oxford, John Radcliffe Hospital, Oxford OX3 9DU, UK; 5 Cardiology Department, Hammersmith Hospital, Imperial College London, London W12 0HS, UK

**Keywords:** myocardial infarction, MRI, coronary physiology, inflammation

## Abstract

**Highlights:**

**What Is Already Known and How This Shapes the Future**

- Highly controlled experimental studies have illustrated the importance of ischaemia time in relation to myocyte necrosis in acute myocardial infarction. However, in humans, the situation is more complex and the relationship between clinical outcomes and ischaemia time is non-linear over a time window that is relevant to clinical presentation.
- Emerging techniques can characterise myocardia in individual patients, providing enhanced understanding of the heterogeneity of the response to ischemic injury.
- Assessments of microvascular function, including secondary changes such as intramyocardial haemorrhaging, should inform decision making regarding additional therapies.
- Early prediction of the nature and extent of myocardial recovery vs. irreversible injury would be useful for prognostic and therapeutic purposes in addition to guiding clinical pathways and safe resource allocation.
- Integrating knowledge of the status of the myocardium and the stages of activation of systemic responses should allow the development and application of therapies based on mechanistic characterisation of upstream signalling pathways and the downstream consequences of effector cells, e.g., of the innate immune system.

**Abstract:**

The past decade has seen a marked expansion in the understanding of the pathobiology of acute myocardial infarction and the systemic inflammatory response that it elicits. At the same time, a portfolio of tools has emerged to characterise some of these processes in vivo. However, in clinical practice, key decision making still largely relies on assessment built around the timing of the onset of chest pain, features on electrocardiograms and measurements of plasma troponin. Better understanding the heterogeneity of myocardial injury and patient-level responses should provide new opportunities for diagnostic stratification to enable the delivery of more rational therapies. Characterisation of the myocardium using emerging imaging techniques such as the T1, T2 and T2* mapping techniques can provide enhanced assessments of myocardial statuses. Physiological measures, which include microcirculatory resistance and coronary flow reserve, have been shown to predict outcomes in AMI and can be used to inform treatment selection. Functionally informative blood biomarkers, including cellular transcriptomics; microRNAs; extracellular vesicle analyses and soluble markers, all give insights into the nature and timing of the innate immune response and its regulation in acute MI. The integration of these and other emerging tools will be key to developing a fuller understanding of the patient-level processes of myocardial injury and repair and should fuel new possibilities for rational therapeutic intervention.

## 1. Introduction

Acute myocardial infarction (AMI) occurs when sustained interruption to the perfusion of the myocardium results in cell death and tissue necrosis. Whilst this is the primary event, it has become increasingly clear that several inter-related processes, which are relevant to myocardial injury and recovery, are set in train by initial ischemic insults. Appreciation of these processes is necessary, since they introduce patient-level heterogeneity that has important implications for selecting effective treatments that optimise benefits and minimise harm. 

Seminal papers by Moroko et al. [1] and Reimer et al. [2] from the 1970s prepared the way for pharmacological and mechanical reperfusion therapies in AMI by showing that in dog experimental models, coronary occlusion did not result in immediate, irreversible transmural infarction. Reimer et al. observed that “*After 40 min of ischemia, myocyte necrosis was subendocardial but with increasing duration of coronary occlusion, irreversible injury progressed as a wavefront toward the subepicardium.* [2]”. Studying time points from 40 min to 24 h after arterial occlusion, they found progressive extension of infarction. The authors concluded that they had identified the “*presence of a subepicardial zone of ischemic but viable myocardium which is available for pharmacologic or surgical salvage for at least three and perhaps six hours following circumflex occlusion in the dog.*” [2]. 

These studies, under highly controlled experimental conditions, have illustrated the importance of time in relationship to myocyte necrosis and shaped current approaches to the management of AMI in humans. Pharmacological and mechanical reperfusion studies have focused on early restoration of flow in the coronary arteries [3]. Duration of ischaemia and door-to-balloon time have even become measures of the quality of health care provision, with substantial investments to reduce the time taken to reach the catheterisation laboratory. Nonetheless, no single parameter, including “ischemia-time”, is likely to accurately determine the optimal management of the complex pathophysiological processes of AMI. 

Advances in technology have enabled in vivo characterisation of injured myocardia in humans. This includes the extent and distribution of acute infarct, microvascular competence, potential for recovery and processes related to inflammation and repair. All these features are increasingly recognised to have important roles in patient outcomes. Nonetheless, the knowledge of these characteristics is poorly utilised in the contemporary management of AMI, where early management decisions are still largely guided merely by the durations of symptoms and features of electrocardiograms. Here, we will highlight the limitations of this relatively crude approach and review how new technical advances have provided novel insights into underlying disease processes, the knowledge of which could bring much-needed precision to clinical decision making, resulting in improved outcomes of reduced risk to individuals. 

## 2. Current Caveats in the Acute Management of Myocardial Infarction

**Figure 1:**
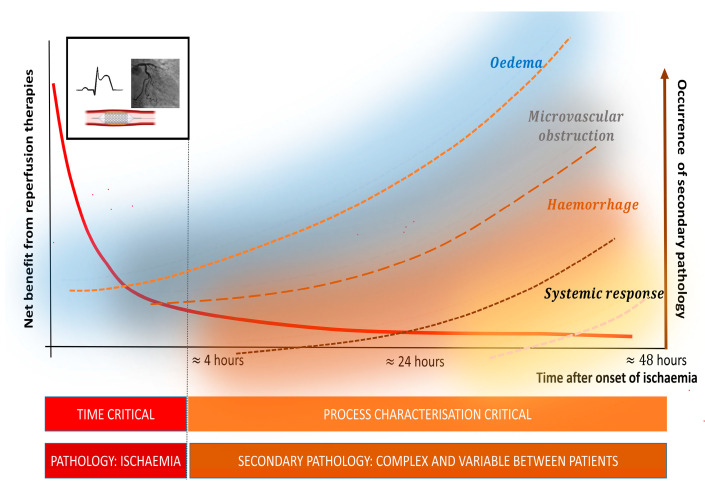
Heterogeneity of pathology in acute myocardial infarction. In the first 3–4 h after the onset of ischaemia, the primary pathology is ischaemia. Accordingly, the emphasis for treatment during this early period is on reperfusion. Delays to treatment are associated with reductions in clinical benefit. Even within those first hours, additional secondary pathological processes begin. These occur because of ischaemia, but they are not amenable to modification by its relief. Moreover, some of these processes, e.g., intramyocardial haemorrhaging, can even be exacerbated by treatments such as fibrinolysis. These heterogenous processes lead to considerable differences in the active processes of injury and repair and variability between patients.

Since AMI is usually caused by an occlusive thrombus on ruptured or eroded atherosclerotic plaque, treatments have been developed, with the aim of restoration of distal perfusion, using either fibrinolytic drugs or percutaneous coronary intervention. Clinical trials of fibrinolytic agents were consistent with the early dog studies and showed that the maximal net benefit from reperfusion therapy would occur within the first 2–3 h of ischaemia, as timed from the onset of symptoms (Figure 1) [4]. This knowledge has driven pharmacological and catheter-based coronary interventions to minimise delays to reperfusion, with the presumed duration of ischaemia assuming prominence in decision making [3].

However, as shown in Figure 1, the relationship between the probability of death and the ischaemia time (defined as the time from symptom onset to reperfusion therapy) is non-linear. The net survival benefit from opening the occluded artery is crucially related to the time from the onset in the very early course of STEMI presentation, but after about 3–4 h, the relationship will change. In this context, the time from the symptom onset is intended as a proxy for the duration of ischaemia and, by extrapolation, for the status of the myocardium. However, at an individual patient level, there are several important caveats. Firstly, the situation in human AMI is more complex than in experimental infarction in dogs, since the time from the symptom onset relies on accurate recollection and does not take into account individual variations such as intermittent or partial coronary occlusion, the extent of collateral circulation, the degree of ischemic preconditioning or the metabolic status of the ischemic myocardium [5]. Secondly, important pathophysiological processes that are not directly related to ischaemia will have been set in motion (Figure 1). For instance, intramyocardial haemorrhaging is a common feature in AMI [2,6]. Haemorrhaging is likely to be exacerbated by fibrinolytic therapies, particularly in patients in whom infarction processes are already relatively advanced [7]. The potential net benefit is likely to be diminished by unselected administration of fibrinolytic therapies and may cause harm in certain subsets [4,7]. Small vessel dysfunction due to endothelial oedema, platelet plugging, pericyte activity or remote and infarct zone inflammation is also invisible to current diagnostic approaches [8,9,10,11]. Similarly, neutrophil and monocyte infiltration have roles in tissue injury and repair but are not characterised at all in the current clinical paradigms [10,12,13,14].

**Figure 2:**
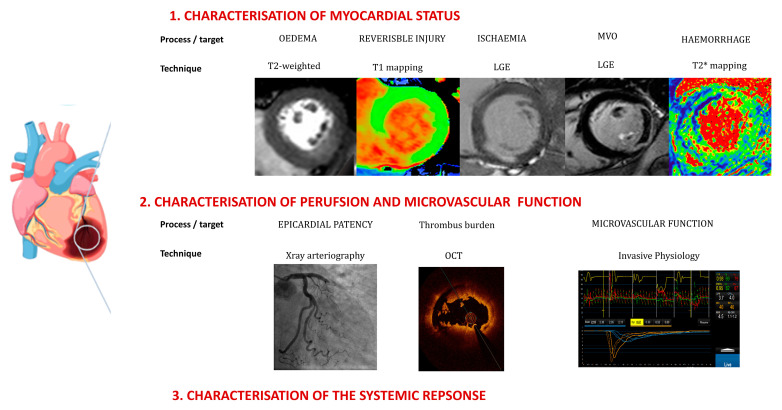
Emerging tools to characterise acute myocardial infarction. New and emerging techniques to identify elements of heterogeneity have focused on (1) characterisation of the myocardium and the consequences of primary ischemic injury and secondary responses; (2) perfusion in relation to the epicardial artery and (3) the systemic inflammatory response.

Approaches to management have emerged, reflecting the well-worn adage that “*time is muscle*” or “*time is brain*” when managing patients with acute MI or ischaemic stroke [15]. However, decision making that is based on the duration of pain, with additional guidance from ST segments on an ECG, is increasingly being seen as an unhelpful oversimplification. Over the past decade, an array of complementary techniques and technologies that can characterise the ischaemic myocardia in individual patients has emerged. Broadly, these can be categorised into (i) blood biomarkers including cellular “*-omics*” [16,17], microRNAs, extracellular vesicle analyses and soluble markers [18]; (ii) direct myocardial characterisation with imaging, especially cardiac MRI [19]; and (iii) physiological measures of microvascular function and integrity [20]. Integrating these emerging techniques in combination with currently used tools could provide a much more precise characterisation of patient statuses that will be necessary for a more personalised approach to the management of AMI (Figure 2). 

## 3. Infarct Distribution

Histological studies have provided important insights into infarct distribution, both in lateral and transmural extension [2,21]. In completed AMI, the extent of myocyte necrosis to the lateral margins is sharply demarcated and follows the anatomical boundaries of the zone subtended by the occluded coronary artery [2,21]. The importance of identifying patients with subendocardial versus transmural infarcts was recognised early, as the former has been associated with better prognosis and functional recovery following revascularisation [22,23]. In clinical practice, identification of transmural necrosis that has already been established (or would inevitably occur) would be useful in the characterisation of AMI and could aid decision making away from redundant or potentially harmful interventions. 

The presence or absence of collateral flow, among other heterogeneous factors such as the metabolic status and the preconditioning of the myocardium, contributes to the distribution of infarction in humans [2,5,24,25]. Therefore, the extent of myocardial infarction will reflect a combination of both the “ischemic time” and patient-level factors that will determine the vulnerability of the myocardium to ischemic injury [2,5,24]. Subendocardial and transmural infarcts can be detected using in vivo imaging techniques (notably MRI), which can provide a platform to understand and characterise the status of the myocardium and the progression of injury at clinically relevant times in AMI in humans. 

The presence of “Q” waves on an ECG is often used as an indication of the transmurality (sometimes termed the “completion”) of AMI and to guide early intervention therapies in those with non-Q-wave AMI [26]. However, this approach is unreliable, and cardiac MRI has demonstrated a large discordant rate of 25–30% between Q waves on the ECG and the extent of transmural MI determined using late gadolinium enhancement [27,28]. Whilst patients who have presented Q waves have had larger infarct sizes, the presence of Q waves has not been associated with less reduction in infarct size after lytic therapy [29]. Similar findings have been reported in the era of primary percutaneous coronary intervention [30,31]. Importantly, the presence of Q waves can be dynamic, and up to 40% of patients have had Q-wave regression after 24 months and as early as 1 h after AMI [31,32]. 

Sometimes, at infarct edges, a mixture of necrotic tissue and viable myocardium exists as isolated islands or peninsulas, and this area has been termed the “border zone” [33]. The distribution of this zone has been linked to the presence of coronary collaterals and is considered an arrhythmogenic substrate that follows AMI [21,33,34]. Such areas are potentially detected as less intense or with lower signals using gadolinium- or oedema-based MRI techniques. This phenomenon was also observed using quantitative methods that measured extracellular volume (ECV) at the infarct borders [21,35,36]. Interestingly, differences in the ECV between the infarct core and its periphery were less evident after one week, reflecting a more homogeneous infarct, and this may suggest a reduction in oedema and remodelling of the infarcted area over time [35,37]. In fact, infarct heterogeneity or “patchiness”, defined using gadolinium-based imaging, has been recognised as a predictor of ventricular arrhythmia and mortality in patients following AMI [38,39]. For every 10% increase in the size of a peri-infarct zone, there was a 25% increase in the hazard of death, even after multiple adjustments for known risk markers [38]. Patients with large peri-infarct areas and non-severe left ventricular (LV) functions had similar prognoses to those with severe LV functions, highlighting the potential utility of using infarct heterogeneity to characterise AMI patients beyond standard imaging parameters [38,40]. 

## 4. Myocardial Characterisation

Changes in the structure of the myocardium, including at the cellular level, are not uniform in response to prolonged ischaemia or reperfusion injury [41,42]. In other words, not all ischaemic myocytes progress inevitably to necrosis. Cellular swelling, secondary to increased membrane permeability and the failure of cell volume regulation that leads to rupture, is the main mechanism of myocyte necrosis [21,42,43]. This subsequently leads to expansion of extracellular volume/water content, which is also exacerbated by other factors, such as reactive hyperaemia and leakage from damaged capillaries [21,42,43]. Therefore, quantifying water content/extracellular volume could help to identify reversible versus irreversible myocardial injury. 

Volumetric changes within the structure of the myocardium, including the distribution of water, has enabled the use of late gadolinium enhancement (LGE) as an in vivo surrogate of infarct size [19,44,45,46]. In circumstances where extracellular space is expanded, such as AMI or chronic MI wherein myocytes are replaced with collagen fibres, gadolinium can accumulate [47,48,49]. Recent development of quantitative MRI mapping techniques has allowed in vivo measurement of ECV and provided further insights into the distribution of acute LGE. Significantly, the ECV in ischemic but salvageable myocardia is significantly different from that in both normal (remote) and infarcted myocardia, permitting its identification using gadolinium-based techniques [50,51]. Although, in the context of coronary disease, LGE has become almost synonymous with infarcted myocardia, this relationship will break down in the early phases of AMI, where LGE characteristics can be identified in myocardia that are not infarcted and subsequently recover. In a rat model of AMI, LGE overestimated infarct size on histology, and showed that gadolinium was present in both infarcted (non-viable) and salvageable (viable) myocardia [52,53]. These findings were corroborated in human studies, where LGE myocardia were significantly, acutely larger compared with those of the same patients at follow-up [46,51,54]. Mechanistically, the washout of gadolinium is related to microvascular function, and impaired microvasculature has been associated with less regression in infarct size [55]. In other words, the ambiguity of LGE on CMR in the acute setting may be, at least in part, related to microvascular dysfunction [55].

Importantly, microvascular dysfunction can be quantified invasively at the time of coronary angiography through the application of pressure/flow guidewires and Doppler thermodilution techniques [56,57]. Both indices are pathologically increased in patients with myocardial injury and coronary microvascular dysfunction and have been extensively validated in patients with AMI, showing strong associations with microvascular obstruction (MVO) on MRI [58,59], with major clinical outcomes [60,61,62].

Oedema-sensitive imaging (such as T2-weighted sequences) offers the ability to identify a volume of myocardium that has recently been subject to significant ischaemia, even when normal perfusion has been subsequently restored (either spontaneously or after treatment). In some sense, oedematous myocardia bear the memory of ischaemia. Thus, they can be used to determine the “volume at risk”. To date, this application has remained largely confined to calculating myocardial salvage indices, with limited data on its prognostic value [63,64,65,66]. The introduction of quantitative techniques, such as T1 and T2 mapping, has overcome some of the limitations related to the use of image analysis to estimate oedema and the imprecision of LGE in the early phases of AMI, as described above. 

Crucially, T1 mapping techniques have enabled us to differentiate reversible versus non-reversible myocardial injury acutely following MI [45]. Cut-off values have been described for normal, salvageable and necrotic myocardia, with an overall accuracy of 97% [45]. Importantly, the proposed threshold for infarcted myocardium has had excellent agreement with infarct sizes at 6 months and has provided accurate information about the extent of infarcts during the acute stage of MI [45]. More recently, we have shown that the average hyperacute (within 3 h of PCI) T1 value within an ischaemic myocardium is able to predict infarct size at 6 months [44]. Such early assessment using T1 mapping could allow precise and direct evaluation of myocardial tissue in response to ischaemia–reperfusion injury before secondary processes can supervene. Additionally, this could enable specific tailored therapies early after primary percutaneous coronary intervention by discerning where irreversible myocardial injury has or has not already occurred. New imaging biomarkers, such as the post-contrast T1 value and ECV (calculated using the ratio of T1 values pre- and post-contrast) may overcome some of the technical limitations associated with using native T1 mapping [67,68]. These markers may help characterise injured myocardia and complement LGE in predicting infarct healing [69,70,71,72].

Recently, a novel quantitative imaging biomarker of microvascular function was systemically used to assess the extent of acute infarction [73]. The absolute rest myocardial blood flow (MBF) was significantly reduced in infarcted compared with remote or salvageable myocardia [73]. The MBF also provided incremental prognostic value in estimating true infarct sizes and functional recovery during the acute setting [73].

Lastly, troponin is a widely used clinical biomarker as a surrogate of infarct size but peaks only after injury has occurred. Furthermore, after successful revascularisation, a rapid washout can produce a misleadingly high peak (but narrow profile) that does not relate to the long-term outcome [74]. While 72 h troponin or total area under the troponin versus time curve may relate more closely to infarct size [75], each requires the passage of time and cannot, therefore, assist early decision making. 

In a clinical setting, early uncertainty about whether a myocardium will recover from an ischemic insult or not will constrain the scope of tailored therapy. Thus, novel imaging biomarkers of the sort described above could provide opportunities to characterise patients and identify those with reversible myocardial injury. The overall emphasis is a move away from a one size fits all approach and estimation of infarct progression based only on the presumed time of ischaemia. Characterising the myocardium, including early indications of reversible vs. irreversible injury (within 3 h of primary PCI), is of prognostic relevance and may set the stage to test new therapeutic options in better stratified populations. 

## 5. Infarct Complexity and Secondary Processes

Microvascular dysfunction is also a major challenge and various techniques have highlighted adverse outcomes in patients with suboptimal microcirculation function [62,76,77]. Prolonged coronary occlusion leads to changes in capillary beds, with increased permeability and higher pressure in the interstitial space extrinsically compressing the intravascular space [21,78]. This microvascular obstruction (MVO) is also exacerbated by endothelial oedema, distal embolisation related to percutaneous coronary intervention and microvascular fibrin-rich thrombosis [21,78,79].

Additional complexity can occur when the failure of an endothelial barrier leads to an intramyocardial haemorrhage (IMH) [21,78,79,80]. Management of both MVO and IMHs is very challenging, and there are no proven therapies to reduce the incidence or extent of either. Importantly, fibrinolytic and intensive anti-thrombotic therapies may exacerbate myocardial injury associated with MVO. This should not be surprising given the complex interactions of clinical and procedural factors that contribute to the development of MVO and IMHs. In a small study of 41 patients, low-dose intracoronary lytic therapy that targeted fibrin-rich coronary thrombosis following primary PCI improved microvascular function [81]. However, low-dose fibrinolytic therapy with alteplase during primary PCI in the T-TIME trial did not reduce MVO, and, in fact, this was associated with increased extents of IMHs in patients with longer ischaemia times [7,82]. Upfront stratification using direct measures of myocardial injury, such as IMR, may allow the identification of suitable patients or the exclusion of those who may be at risk of harm with particular therapies. Compared to cardiac MRI-derived MVO, which mainly provides an anatomical evaluation of the actual area of the injured myocardium, both hMR and IMR depict the functional status of the coronary microvascular bed subtended by the infarct-related artery. Importantly, each can be obtained during the revascularisation procedure and can potentially inform decision making in real time. On the other hand, both IMR and hMR relate to the combined necrotic/infarct core plus the peri-infarct watershed zone. It could be argued that this does not allow sufficient functional discrimination between the two compartments of a jeopardised myocardium subtended by an infarct-related artery. This lack of spatial definition might also partially explain the relatively weak correlation between hMR/IMR and the extent of MVO. There is a discordance between IMR and MVO reported in more than one-third of cases, i.e., high IMR with no MVO or MVO with low IMR [55]. A temporal difference in acquiring these data could also account for such a divergence, since IMR and hMR are acquired in the very acute phase, right at the time of a coronary angiogram, in the catheterisation laboratory, whilst MVO is obtained from MRI and is usually obtained a few hours or even days after initial admission with AMI. Nonetheless, it has been shown that functional coronary microvascular dysfunction (defined as IMR > 40) can characterise a worse degree of MVO, in terms of both extension and the concomitant presence and degree of IMHs. These observations also provide an insight as to why patients with preserved functional coronary microcirculation proceed to better left-ventricle remodelling, with more regression in their LGE myocardia [55].

Carrick et al. showed that all patients with IMHs in their study also had evidence of MVO [37,83]. Therefore, it is plausible that MVO precedes an IMH and the absence of MVO would eliminate or markedly reduce the chance of developing IMHs. Whilst ischaemia time is a strong predictor of IMHs and MVO, their presence is not inevitable in late-presentation AMI. Importantly, both MVO and IMHs are significant predictors of adverse clinical outcomes, including death and re-admission with congestive cardiac failure, independently of infarct size [77,84]. Notably, IMHs seemed to be more closely related to adverse clinical outcomes than MVO, and patients who developed both were associated with even worse clinical outcomes [83]. Clearly, the knowledge that these pathological processes occur in some patients but not others and their association with adverse outcomes raises questions about how they might be ameliorated (or exacerbated) with treatment selection. Accordingly, De Maria et al. developed a scoring tool to predict patients with high likelihoods of developing MVO and IMHs [85,86]. Those authors demonstrated that elderly patients who present large thrombus burdens and high pre-stenting IMR have larger extents of MVO and IMHs [85]. Notably, this scoring system allowed early determination in the catheterisation laboratory at the start of the procedure, making it possible to test and triage additional/alternative and novel therapeutic options in high-risk patients in the very early stages of the revascularisation procedure [85]. In this regard, the OxAMI-PICSO study was the first to use IMR to guide selection of additional therapy for patients with AMI. The OxAMI-PICSO study showed how additional therapy with pressure-controlled intermittent coronary sinus occlusion (PICSO) was effective in reducing the extent of the infarct size in selected anterior MI patients with pre-procedural IMR values of above 40 units. Moreover, the extent of the infarct size at 6 months was comparable between patients who received PICSO and those with pre-procedural IMR values of below 40 units, although the latter group did not receive the additional treatment on top of conventional coronary revascularisation [87].

## 6. Infarct Recovery

Infarct recovery is a complex process that is related to not solely the initial insult of interrupted perfusion but also secondary processes that are variable in different patients, including inflammatory cellular infiltration, the resorption of oedema, the replacement of necrotic myocytes with fibrous tissue and angiogenesis [88]. Even remote inflammation in non-ischemic myocardia plays a major role in infarct healing [10,89]. The previously presumed “static” nature of ischaemic myocardia is no longer accepted, and the dynamic changes of injured myocardia have brought into question the utility of one-off imaging-based assessments of treatment efficacy [90,91]. MRI has been used as a tool to allow retrospective assessment of ischaemic myocardia and applied in the evaluation of the efficacy of new treatments using the myocardial salvage index. However, the use of this method relies on the assumption that the extent of an ischaemic myocardium (or area at risk) is stable during the early period after AMI [46,92]. Early studies showed reductions in myocardial oedema during the second week post-AMI [46]. More recently, experimental and human data have suggested that oedema is not stable and will undergo a bimodal pattern during the first week [37,93,94]. The initial wave will peak within few hours and diminish after 24 h and has been attributed to reperfusion injury [37,93,94]. The second wave will start at day 3 and peak after a week and has been linked to the inflammatory responses during myocardial healing processes [37,93,94]. Importantly, the resolution of the first wave was attributed to its intensity but not its extent (area of the abnormal myocardium), which was static within the first week [37]. This variability adds challenges in calculating the myocardial salvage index, given the dynamic nature of ischaemic myocardia, and, consequently, will make the assessment of future cardio-protection studies imprecise. Therefore, a recent consensus was developed to define optimal timing and proposed performance of MRI between days 3 and 7 following AMI [21].

Using quantitative T1 and T2 mapping techniques, new insights into infarct recovery have also been obtained. Absolute T1 values at 6 months have remained relatively higher in areas of previous infarction, but not in salvageable myocardia. These areas of high T1 values likely reflect the replacement of myocytes with fibrous tissue [45]. Similarly, T2 values remained higher in the infarcted compared with the remote myocardia at 6 months following AMI [95]. Persistently high T2 values were noted in two-thirds of patients and were more likely to occur in patients with large infarcts and occluded vessels at the outset [95]. This also reflected worse left-ventricle remodelling and was related to increased risks of mortality and heart failure, although the relative change in T2 values was better related to adverse clinical outcomes [95]. Persisting oedema or fat replacement may account for these changes in T2 values at follow-up [95,96]. Human studies have suggested that MVO is stable over the first 48 h and will subsequently diminish over the next few weeks post-AMI [37,97,98]. The extent of MVO has been directly linked to increased mortality, and for every 1% increase in MVO as a percent of the left-ventricle mass, there is a 9% increased risk of mortality, even after adjustment for infarct size [77]. Residual iron deposits secondary to IMH breakdown at follow-up have been detected with MRI and validated using histology [99,100]. Residual iron deposits were, subsequently, confirmed in multiple imaging studies and have been linked to inflammation, adverse remodelling and worse clinical outcomes [100,101]. This “chronic” marker of microvascular injury could be used to identify high-risk patients that may benefit from further specific post-infarction therapy.

Besides MRI-based observations, the measurement of invasive indices of coronary physiology has also provided similar evidence of functional recovery of coronary microcirculation, depicted as a progressive normalisation of vasodilatory capacity in response to vasodilating agents such as adenosine. 

Revascularisation itself can affect coronary microvascular vasodilatory functions both positively and negatively, as reflected by a change in the IMR. Approximately 40% of patients have shown incomplete recovery or even worsening of microvascular functions after revascularisation [102]. A large atherothrombotic burden (identified using optical coherence tomography), prolonged ischaemic time and a pre-existing severe degree of coronary microvascular impairment are all predictors of incomplete recovery and/or of the deterioration of coronary microvascular function in the subacute phase post-AMI [103].

Cuculi et al. [104] have gone further and reported that the process of recovery of coronary microvascular function continues in the chronic phase after AMI. Those investigators described a progressive reduction in coronary microvascular resistance (depicted with IMR) during the 6 months after the acute event. This reduction was mirrored by a reduction in the fractional flow reserve (FFR) in the infarct-related artery [104]. Notably, this favourable evolution in IMR and the FFR was, however, blunted in patients with evidence of anatomical microvascular injury (e.g., MVO) in MRI scans performed at 48 h after the acute event. Similar findings were also reported using non-invasive assessment of resting MBF, with markedly less improvements in areas that had previous MVO or IMHs [73].

## 7. Response to Infarction

**Figure 3:**
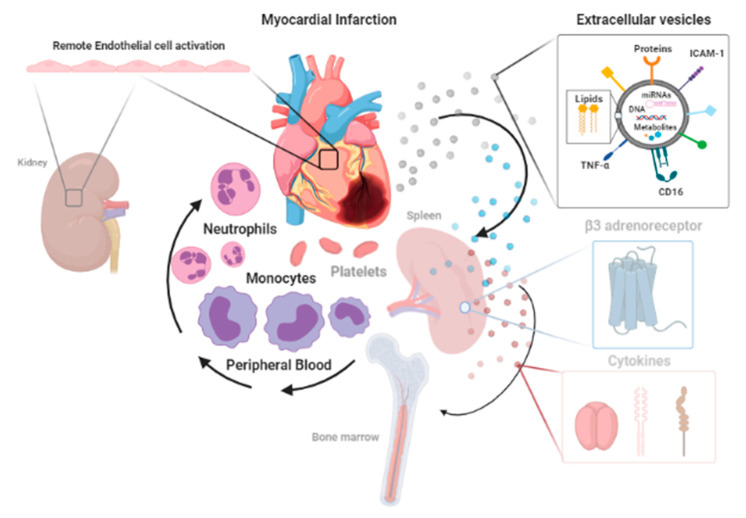
Functional blood biomarkers that provide insight into the innate immune response and its regulation in acute myocardial infarction. The ischemic myocardium signals its injured state through “afferent” pathways that include cytokines, extracellular vesicles and miRNA content and by activation of the sympathetic nervous system. The “efferent” response involves the mobilisation and transcriptional/functional activation of immune cells from remote reserves. The characterisation of each limb has the potential to provide detailed information on the stage and nature of the systemic inflammatory response.

A vigorous inflammatory cell response is a cardinal feature of AMI. Studies in animal models have shown that aspects of cellular inflammation are involved in the propagation of injury as well as its resolution, its repair and scar formation [10,89,105,106]. As shown in Figure 3, diagnostic tools that contribute to the mechanistic understanding of the systemic response are likely to include complementary characterisation of upstream signalling pathways and the downstream consequences in different effector-cell types. 

Neutrophils are the first cells to increase in peripheral blood following AMI [107,108]. In animal models, inhibition of neutrophil recruitment in AMI can reduce infarct size [109,110]. Monocytes are also a potential focus for therapy following AMI because they contribute to the proinflammatory phase in the immediate days following injury [111]. In the acute phase, infiltrating cells scavenge necrotic debris and are active in local proteolysis and phagocytosis [12]. Simultaneously, the adaptive immune system is activated and plays roles in the further release of proinflammatory cytokines and reduction in the levels of circulating anti-inflammatory cytokines [112,113].

Systemic activation is evidenced by the increased plasma levels of circulating inflammatory cytokines [114] and endothelial activation at remote sites [115]. The release of a number of proinflammatory mediators (e.g., interleukin (IL)-1β, tumour necrosis factor (TNF)-α and IL-6) and endothelial cell-derived extracellular vesicles (EVs) promotes and facilitates the recruitment of inflammatory cells (e.g., neutrophils, monocytes) from the blood and also from remote sites, including the spleen and bone marrow [111]. 

EVs are submicron-sized lipid envelopes that carry surface proteins, which enable receptor-mediated signalling with cells locally in tissues or distally from their sites of origin through liberation into peripheral blood. Plasma EVs are elevated at the time of presentation of AMI and correlate with the extent of myocardial injury as determined with T2W MRI [111]. Plasma EVs are a heterogenous pool, but, following AMI, there is significant enrichment for endothelial cell-derived EVs (EC-EVs), defined by the presence of VCAM-1 in their membranes. Furthermore, in AMI, plasma EVs are enriched for a small number of miRNAs, including endothelial-cell-associated miRNAs-126-3p and miRNA-126-5p. Significantly, these EC-EVs activate transcriptional programmes in remote monocyte reserves and induce pathway activation comparable to the responses observed in peripheral blood monocytes following AMI in humans [10].

The expanding understanding of the means by which the ischemic myocardium signals to remote reserves and the appreciation that responding leukocytes show particular patterns of transcriptomic response opens the possibility to map the systemic inflammatory statuses of patients in response to MI with increasing precision and to combine these with the characterisation of the status of the myocardia themselves. With greater understanding of the processes described above, it may be possible to augment current tools to gain a better understanding of patient-specific clinical statuses, far enhancing currently used biomarkers such as the ECG and serum troponin [116]. The measurements of novel proteins that reflect underlying activated biological pathways [117], the determination of circulating miRNAs [118], the characterisation of EVs [119] and profiling of the transcriptomes of key cell types are likely to identify specific biological pathways [10,120,121] and guide the precise utilisation of newer biological agents (e.g., IL-1R antagonists [122], TNF antagonists [123]) that may only benefit small groups of patients whilst avoiding unnecessary risks and associated economic costs in others [89,124].

**Figure 4:**
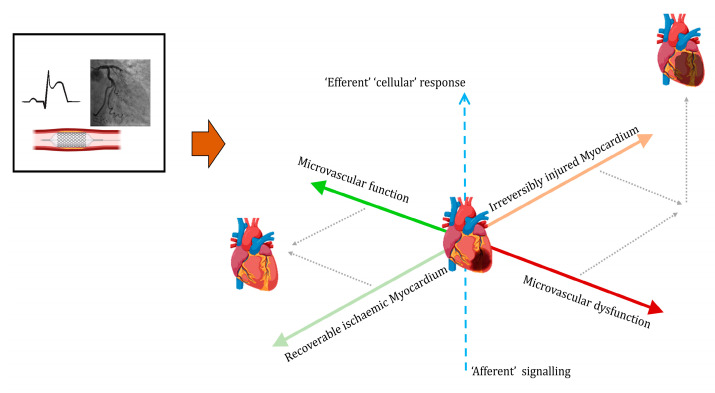
Integrated application of complementary approaches. Schematic to highlight the potential for diversity. In current clinical treatment pathways for acute ST segment elevation, MI is determined with clinical presentation, including duration of chest pain and the ECG. A coronary angiogram can identify the occlusion of a major epicardial artery treated with stent implantation and attendant anti-platelet drugs. Relevant secondary processes, some of which begin even before reperfusion therapy, play little or no part in patient stratification and decision making, even though they can vary widely in patients with very similar modes of presentation and early initial treatments. The green bubble represents a patient with successful PCI and no secondary adverse consequences. The brown bubble represents a patient with established infarction; microvascular dysfunction and activation of systemic inflammatory responses.

As illustrated in Figure 4, an integrated approach to patient characterisation that incorporates measurements of myocardial status; microcirculatory competence and the nature/stage of the inflammatory response stands to delineate disparate patient groups. 

## 8. Conclusions

With better understanding of underlying biological processes and technological advances to rapidly profile clinical samples, it may be possible to diagnose patients sooner, obtain mechanistically relevant characterisation, stratify the patients’ risk, institute specific bespoke therapies and obtain a better understanding of prognosis. These tools and their successors therefore have the potential to improve care from the current “one size fits all” approach to a more personalised approach, improving safety and reducing risks, thereby ultimately improving clinical outcomes.

## Data Availability

Not applicable.
